# Donor Time to Death and Kidney Transplant Outcomes in the Setting of a 3-Hour Minimum Wait Policy

**DOI:** 10.1001/jamanetworkopen.2024.43353

**Published:** 2024-11-14

**Authors:** Samuel J. Tingle, Nicholas D. H. Chung, Abdullah K. Malik, Georgios Kourounis, Emily Thompson, Emily K. Glover, Jennifer Mehew, Jennifer Philip, Dale Gardiner, Gavin J. Pettigrew, Chris Callaghan, Neil S. Sheerin, Colin H. Wilson

**Affiliations:** 1 National Institute of Health and Care Research Blood and Transplant Research Unit, Newcastle University and Cambridge University, Newcastle upon Tyne, United Kingdom; 2 Translational and Clinical Research Institute, Newcastle University, Newcastle upon Tyne, United Kingdom; 3 Institute of Transplantation, Freeman Hospital, Newcastle upon Tyne, United Kingdom; 4 Renal Services, Newcastle upon Tyne Hospitals NHS Foundation Trust, Newcastle upon Tyne, United Kingdom; 5 Statistics and Clinical Research, NHS Blood and Transplant, Bristol, United Kingdom; 6 Division of Transplantation, University of Wisconsin School of Medicine and Public Health, Madison; 7 Deceased Organ Donation, NHS Blood and Transplant, Bristol, United Kingdom; 8 Consultant in Adult Intensive Care Medicine, Nottingham University Hospitals NHS Trust, Nottingham, United Kingdom; 9 Department of Surgery, University of Cambridge, Cambridge, United Kingdom; 10 Department of Nephrology and Transplantation, Guy’s Hospital, Guy’s and St Thomas’ NHS Foundation Trust, London, United Kingdom

## Abstract

**Importance:**

Lengthening waiting lists for organ transplant mandates the development of strategies to expand the deceased donor pool. Due to concerns regarding organ viability, most organ donation organizations internationally wait no longer than 1 to 2 hours for potential donation after circulatory death (DCD), possibly underutilizing an important organ source; UK policy mandates a minimum 3-hour wait time.

**Objective:**

To assess whether time to death (TTD) from withdrawal of life-sustaining treatment (WLST) is associated with kidney transplant outcomes.

**Design, Setting, and Participants:**

This population-based cohort study used data from the prospectively maintained UK Transplant Registry from all 23 UK kidney transplant centers from January 1, 2013, to December 31, 2021; follow-up was until the date of data extraction (October 2023). Participants comprised 7183 adult recipients of DCD kidney-alone transplants.

**Exposure:**

Duration of TTD, defined as time from WLST to donor mechanical asystole.

**Main Outcomes and Measures:**

Primary outcome was 12-month estimated glomerular filtration rate (eGFR; for the main eGFR model, variables with significant right skew [histogram visual assessment] were analyzed on the log_2_ scale), with secondary outcomes of delayed graft function and graft survival (censored at death or 5 years).

**Results:**

This study included 7183 kidney transplant recipients (median age, 56 years [IQR, 47-64 years]; 4666 men [65.0%]). Median donor age was 55 years (IQR, 44-63 years). Median TTD was 15 minutes (range, 0-407 minutes), with 885 kidneys transplanted from donors with TTD over 1 hour and 303 kidneys transplanted from donors with TTD over 2 hours. Donor TTD was not associated with recipient 12-month eGFR on adjusted linear regression (change per doubling of TTD, −0.25; 95% CI, −0.68 to 0.19; *P* = .27), nor with delayed graft function (adjusted odds ratio, 1.01; 95% CI, 0.97-1.06; *P* = .65) or graft survival (adjusted hazard ratio, 1.00; 95% CI, 0.95-1.07; *P* = .92). These findings were confirmed with restricted cubic spline models (assessing nonlinear associations) and tests of interaction (including normothermic regional perfusion). In contrast, donor asystolic time, cold ischemic time, and reperfusion time were independently associated with outcomes. Compared with a theoretical 1-hour maximum wait time, the UK policy (minimum 3-hour wait time) has been associated with 885 extra DCD transplants compared with 6298 transplants (14.1% increase).

**Conclusions and Relevance:**

In this cohort study of DCD kidney recipients, donor TTD was not associated with posttransplant outcomes, in contrast to subsequent ischemic times. Altering international transplant practice to mandate minimum 3-hour donor wait times could substantially increase numbers of kidney transplants performed without prejudicing outcomes.

## Key Points

**Question:**

Is prolonged donor time to death after withdrawal of life-sustaining treatment (WLST) in donation after circulatory death (DCD) associated with kidney transplant outcomes?

**Findings:**

In this cohort study of 7183 DCD kidney transplant recipients, donor time to death was not associated with short-term or long-term kidney transplant outcomes. Compared with a theoretical maximum wait time of 1 hour, the UK minimum wait time of 3 hours after WLST was associated with a 14.1% increase in the number of DCD kidney transplants.

**Meaning:**

This study suggests that organ donation organizations can safely extend the minimum wait time to 3 hours to significantly increase the number of kidneys available for transplant internationally.

## Teaser

This cohort study assesses whether prolonged donor time to death after withdrawal of life-sustaining-treatment is associated with kidney transplant outcomes.

## Introduction

For patients with end-stage kidney disease undergoing dialysis, a kidney transplant offers advantages in survival, quality of life, and health care costs.^1,2^ However, as the global prevalence of chronic kidney disease increases,^3^ transplant waiting times continue to lengthen, with only one-fourth of the individuals listed for transplant in the US receiving a deceased-donor kidney within 5 years.^4^ Strategies that either expand the deceased-donor pool or improve use of organs from existing donors are thus required.

The key to improving use of organs from potential donors is identifying factors that are incorrectly perceived to compromise outcomes. One such potential factor is time to death (TTD) in controlled donation after circulatory death (DCD). Concerns persist that the potential warm ischemic insult from lengthy TTD may affect recipient outcomes. Many organ donation organizations (ODOs) therefore have strict DCD wait times of no more than 1 or 2 hours after withdrawal of life-sustaining treatment (WLST), with only 6.7% of US ODOs routinely waiting even 2 hours.^5,6,7^ Thus, substantial numbers of viable kidneys are potentially lost due to these strict wait times.

With the introduction of the UK National Organ Retrieval Service over a decade ago, the national standard for DCD minimum wait time was set to a minimum of 3 hours^8^ based on a single center’s demonstration that favorable kidney transplant outcomes can be achieved from donors with prolonged TTD.^9^ Thus, the UK has over a decade of experience transplanting kidneys from donors with prolonged TTD. Coupled with high UK DCD donation rates,^10^ this provides a unique dataset to assess how the duration of TTD is associated with outcomes after DCD kidney transplant.

## Methods

This national cohort study was performed using prospectively collected data from the UK Transplant Registry, derived from the 23 UK kidney transplant units. NHS Blood and Transplant granted data access after internal review board approval. NHS Blood and Transplant also determined that study-specific ethical review or approval was not required, nor was study-specific informed consent. We included adult (aged ≥18 years at the time of transplant) recipients of Maastricht criteria III DCD kidney-only transplants performed between January 1, 2013, and December 31, 2021.^11^ Exclusion criteria were multiorgan transplants (including simultaneous pancreas and kidney transplants), dual kidney transplants, and ABO-incompatible transplants or human leukocyte antigen antibody–incompatible transplants (n = 71). Pretreatment of DCD donors with heparin is not performed in the UK. Data were extracted on October 27, 2023. Ethnicity is reported here exactly as provided by the UK Transplant Registry; there are no additional data on the ethnic minorities coded as “other.” Ethnicity is known to affect outcomes in the UK setting, and was therefore required as a confounder to assess the independent association of TTD with posttransplant outcomes. This study followed the Strengthening the Reporting of Observational Studies in Epidemiology (STROBE) reporting guideline.^12^

The primary exposure was TTD, defined as the time from WLST until mechanical asystole. The UK has a 5-minute “hands-off” period after mechanical asystole, after which death is confirmed and DCD donation proceeds. Asystolic time was defined as the time between mechanical asystole and aortic cold flush or start of normothermic regional perfusion (NRP). Definitions of TTD, functional TTD (fTTD), and other ischemic times can be found in eTable 1 in Supplement 1. Time-to-death values over 600 minutes (n = 19) were deemed likely timestamp errors and removed. UK policy states: “Abdominal teams must wait at least three hours for the onset of functional warm ischaemia (defined as systolic BP [blood pressure] <50 mm Hg). If the systolic blood pressure has not fallen <50 mm Hg [at three hours] then they may stand down at that stage.”^8^ Kidney allocation was via a national offering scheme during the years 2019 to 2021, and a hybrid national and local scheme during the years 2013 to 2019. The withdrawal process was identical to that for nondonating persons.^13^

### Outcomes

The primary outcome was recipient 12-month estimated glomerular filtration rate (eGFR; CKD-EPI [Chronic Kidney Disease Epidemiology Collaboration] 2021 formula),^14^ with recipients losing their graft before 1 year given a nominal eGFR value of 10 mL/min/1.73 m^2^.^15^ Secondary outcomes were incidence of delayed graft function (DGF; defined as the need for dialysis in the first week after transplant) and graft survival (censored at death or 5 years). Median follow-up was estimated using the reverse Kaplan-Meier method with graft survival.^16^

### Statistical Analysis

To account for missing data, multiple imputation was performed (aregImpute; Hmisc R package in R, version 4.1.2 [R Project for Statistical Computing]) to generate 20 imputed datasets.^17^ This uses predictive mean matching with bootstrap draws to build rich additive restricted cubic spline models.^18^ This was chosen in preference to multiple imputation by chained equations, as it preserves nonlinear associations. This was especially important as our outcome models use nonlinear modeling. Multiple imputation included variables listed in eTable 2 in Supplement 1. eGFR and DGF were included as variables in the multiple imputation model, to preserve associations between variables and outcome.^19,20^

To assess the association of TTD with 12-month eGFR, multiple linear regression was performed, pooling results from all 20 imputed datasets, adjusting variance based on both within- and between-imputation variation, using the fit.mult.impute function (Hmisc package in R, version 4.1.2).^17^ Adjustment for a wide range of confounders was performed. Potential confounders were selected based on previous research and clinical expertise; statistical variable selection techniques (eg, stepwise selection) were avoided.^21^ NHS Blood and Transplant mismatch groups were used (eTable 3 in Supplement 1).

For the main eGFR model, variables with significant right skew (histogram visual assessment) were analyzed on the log_2_ scale. As the association of TTD with outcomes may differ with donor age, NRP, asystolic or cold ischemic time (CIT), or year, an additional model was built that included interaction terms. Sensitivity analyses were also performed that included additional potential confounders: transplant year, transplant center, machine perfusion (none, hypothermic, or normothermic), and highly sensitized patients (calculated reaction frequency >85%).^22^ Finally, a sensitivity analysis was performed with random intercepts for each donor, to account for the clustered nature of the data. The same model building approach was used for DGF and graft survival (logistic and Cox proportional hazards regression, respectively). These models were repeated with restricted cubic splines used for TTD, ischemic times of interest, and donor age. This avoids assumptions of linear associations and offers greater power than splitting into arbitrary groups. For eGFR and DGF, 5 knots were used (5th, 27.5th, 50th, 72.5th, and 95th percentiles), and 4 knots (5th, 35th, 65th, and 95th percentiles) were used for graft survival (fewer knots to prevent overfitting).^18^

Kaplan-Meier plots and the number of transplants each year from donors with various TTDs were generated using pooled imputed data. All *P* values were from 2-sided tests and results were deemed statistically significant at *P* < .05. All analyses were performed in R, version 4.1.2 (R Project for Statistical Computing)^23^ using the following packages: tidyverse, rms, Hmisc, and survminer.^17,24,25,26^

## Results

**Figure 1.**
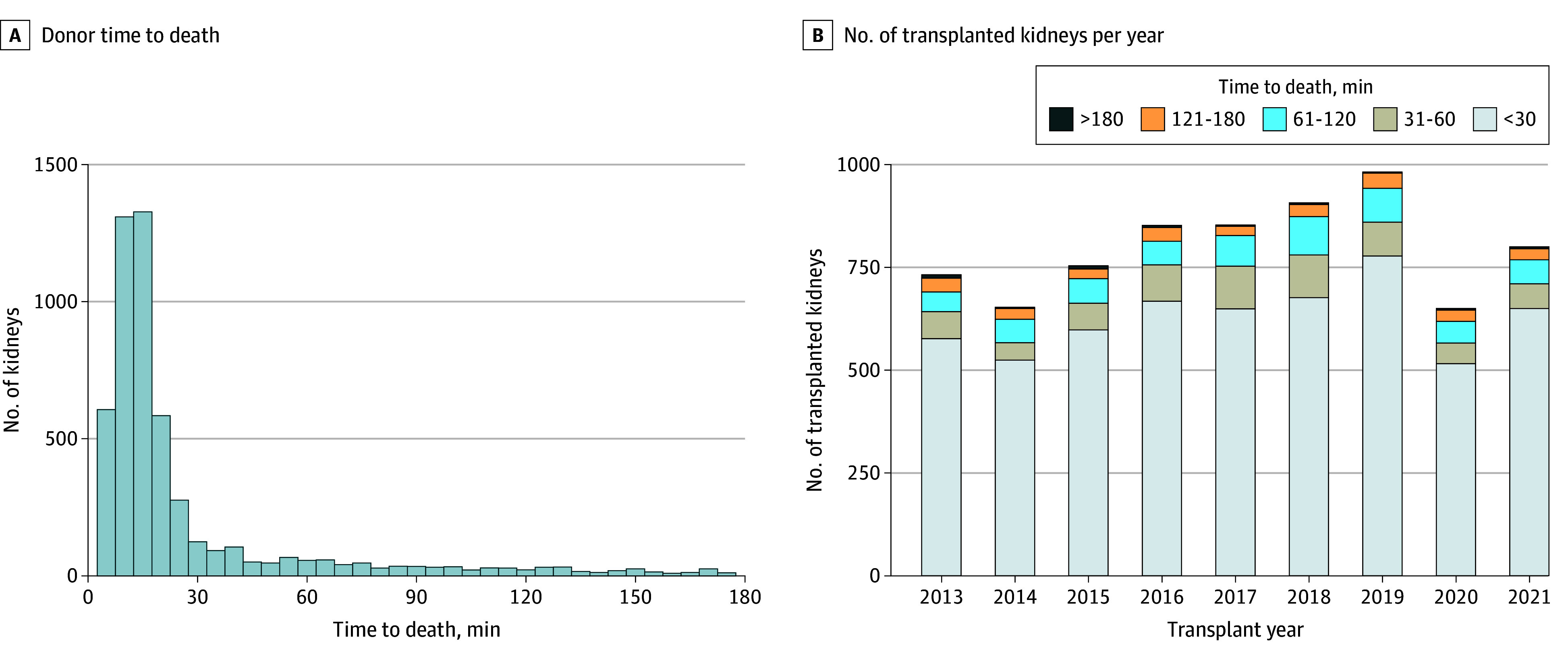
Distribution of Donor Time to Death for Transplanted Kidneys A, Histogram of donor time to death (withdrawal of life sustaining treatment to mechanical asystole) (raw data). B, Number of transplanted kidneys each transplant year categorized by time to death (imputed data).

From UK DCD donors between 2013 and 2021, 7183 kidney recipients (median age, 56 years [IQR, 47-64 years]; 4666 men [65.0%] and 2515 women [35.0%]) from 4102 donors (median age, 55 years [IQR, 44-63 years]) were included in the analysis, with a median follow-up time of 3.9 years (IQR, 1.9-6.0 years) (flow diagram in eFigure 1 in Supplement 1). Key cohort demographics are given in Table 1, with additional demographics and full description of missing data for all variables shown in eTable 2 in Supplement 1. The median TTD was 15 minutes (range, 0-407 minutes). The distribution of TTD values for transplanted kidneys is given in Figure 1A. The number of kidney transplants performed each year from donors with various TTD values is shown in Figure 1B (raw data in eTable 4 in Supplement 1). An estimated 5635 transplants were performed from donors with TTD of less than 30 minutes, 663 from donors with TTD of 30 to less than 60 minutes, 582 from donors with TTD of 1 to 2 hours, 261 from donors with TTD of 2 to 3 hours, and 42 from donors with TTD of over 3 hours (calculated from the multiple imputed datasets). Cohort demographics in these subgroups are given in eTable 5 in Supplement 1.

**Table 1.** Cohort Demographic Characteristics

Characteristic	Patients, No. (%) (N = 7183)
Donor characteristics	
Age, median (IQR), y	55 (44-63)
Sex	
Male	4476 (62.3)
Female	2707 (37.7)
Cause of death	
Hypoxic brain injury	2847 (39.6)
Ischemic stroke	508 (7.1)
Intracranial hemorrhage	2498 (34.8)
Trauma	314 (4.4)
Other	765 (10.7)
Missing	251 (3.5)
Normothermic regional perfusion	
No	6730 (93.7)
Yes	453 (6.3)
Ischemic times, median (IQR), min	
TTD	15.0 (10.0-25.0)
Functional TTD	3.0 (1.0-7.0)
Asystolic time	13.0 (11.0-16.0)
Nephrectomy time	43.0 (32.0-58.0)
CIT	771.0 (600.0-977.0)
Reperfusion time	38.0 (31.0-46.0)
Recipient characteristics	
Age, median (IQR), y	56 (47-64)
Sex	
Male	4666 (65.0)
Female	2515 (35.0)
Primary kidney disease	
Diabetes	925 (12.9)
Glomerulonephritis	1293 (18.0)
Hypertension	474 (6.6)
Polycystic kidney disease	933 (13.0)
Pyelonephritis or reflux nephropathy	391 (5.4)
Other	1292 (18.0)
Missing	1875 (26.1)
Posttransplant outcomes	
eGFR at 1 y after transplant, median (IQR), mL/min/1.73 m^2^	47.4 (33.2-63.3)
Function of kidney after transplant	
Immediate	4333 (60.3)
Delayed	2146 (29.9)
Primary nonfunction	192 (2.7)
Missing	512 (7.1)

Abbreviations: CIT, cold ischemic time; eGFR, estimated glomerular filtration rate (CKD-EPI [Chronic Kidney Disease Epidemiology Collaboration] 2021 formula); TTD, time to death (withdrawal of life-sustaining treatment to mechanical asystole).

### Association With 12-Month eGFR

A multiple linear regression model was used to assess the association of TTD with recipient 12-month eGFR, adjusting for a wide range of factors (Table 2). Donor TTD was not associated with recipient 12-month eGFR; the difference in 12-month eGFR per doubling of TTD was −0.25 (95% CI, −0.68 to 0.19; *P* = .27). In contrast, increasing CIT and reperfusion time (also termed *second warm ischemic time*) were associated with worsening 12-month eGFR.

**Table 2.** Multiple Linear Regression Model for 12-Month eGFR, Pooled From 20 Imputed Datasets (N = 7183)^a^

Variable	Adjusted coefficient (95% CI)	*P* value
Log_2_ TTD	−0.25 (−0.68 to 0.19)	.27
Log_2_ asystolic time	−1.16 (−2.45 to 0.13)	.08
Log_2_ nephrectomy time	0.93 (−0.02 to 1.87)	.05
Log_2_ cold ischemic time	−2.49 (−3.48 to −1.50)	<.001
Reperfusion time, per 10 min	−0.50 (−0.92 to −0.09)	.02
Received NRP	5.60 (3.48 to 7.72)	<.001
Recipient sex: female	−2.06 (−3.14 to −0.98)	<.001
Recipient age, y	−0.08 (−0.13 to −0.03)	.001
Recipient BMI	−0.32 (−0.44 to −0.21)	<.001
Recipient ethnicity		
Asian	5.18 (3.79 to 6.57)	<.001
Black	−6.11 (−7.98 to −4.25)	<.001
Other	1.46 (−2.02 to 4.94)	.41
White	[Reference]	
Primary kidney disease		
Diabetes	[Reference]	
Glomerulonephritis	−0.89 (−2.70 to 0.93)	.34
Hypertension	−0.22 (−2.43 to 2.00)	.85
Polycystic kidney disease	0.68 (−1.28 to 2.63)	.50
Pyelonephritis or reflux nephropathy	−1.82 (−4.36 to 0.72)	.16
Other	−1.53 (−3.32 to 0.26)	.10
Log_2_ recipient wait time, d^b^	−0.55 (−0.90 to −0.21)	.002
Previous kidney transplants, No.		
0	[Reference]	
1	−1.01 (−2.73 to 0.70)	.25
>1	−7.28 (−11.42 to −3.13)	.001
Donor sex: female	−1.11 (−2.18 to −0.04)	.04
Donor age, y	−0.56 (−0.61 to −0.52)	<.001
Donor cause of death		
Hypoxic brain injury	[Reference]	
Ischemic stroke	−2.35 (−4.31 to −0.39)	.02
Intracranial hemorrhage	−2.66 (−3.83 to −1.48)	<.001
Trauma	0.95 (−1.55 to 3.45)	.46
Other	−2.79 (−4.55 to −1.03)	.002
Log_2_ donor creatinine at retrieval	−1.99 (−2.82 to −1.16)	<.001
Donor diabetes status: present	−4.90 (−6.78 to −3.01)	<.001
Donor past history of drug abuse: present	2.60 (1.12 to 4.08)	.001
Donor hypertension: present	−3.55 (−4.73 to −2.37)	<.001
Visual quality of perfusion		
Good	[Reference]	
Fair	−2.78 (−4.35 to −1.21)	.001
Poor	−4.69 (−7.68 to −1.70)	.002
Patchy	−3.36 (−5.74 to −0.97)	.01
HLA mismatch level^c^		
1	[Reference]	
2	−0.39 (−3.27 to 2.49)	.79
3	0.72 (−2.06 to 3.50)	.61
4	0.38 (−2.63 to 3.38)	.81

Abbreviations: BMI, body mass index; eGFR, estimated glomerular filtration rate; HLA, human leukocyte antigen; NRP, normothermic regional perfusion; TTD, time to death (withdrawal of life-sustaining treatment to mechanical asystole).

^a^ Right-skewed variables are log_2_ transformed, so the results relate to the change in 12-month eGFR every time the variable doubles. A nominal eGFR value of 10 mL/min/1.73 m^2^ was used for those with graft failure before 1 year.

^b^ Wait time from becoming active on the transplant waiting list.

^c^ For HLA mismatch level details, see eTable 3 in Supplement 1.

We hypothesized that the association of TTD with outcomes may differ based on other donor or transplant factors. This hypothesis was tested by adding interaction terms to the model in Table 2. These additions revealed no evidence that the association between TTD and eGFR varies based on use of NRP (interaction coefficient, –0.19; 95% CI, –1.87 to 1.49; *P* = .82 for interaction), increasing donor age (interaction coefficient, 0.01; 95% CI, –0.02 to 0.04; *P* = .51 for interaction), asystolic time (interaction coefficient, –0.57; 95% CI, –1.68 to 0.54; *P* = .31 for interaction), CIT (interaction coefficient, 0.43; 95% CI, –0.38 to 1.23; *P* = .30 for interaction), or year of transplant (interaction coefficient, 0.03; 95% CI, –0.13 to 0.18; *P* = .74 for interaction). Sensitivity analyses were also performed including the following additional confounders to the model in Table 2: year of transplant, use of machine perfusion, recipient hospital, and highly sensitized patients. Results of these sensitivity analyses were all consistent with our main results. Adding random intercepts for each unique donor (to account for clustering) was also consistent with the main results, with no clinically relevant changes to TTD or ischemic time effect estimates or 95% CIs.

**Figure 2.**
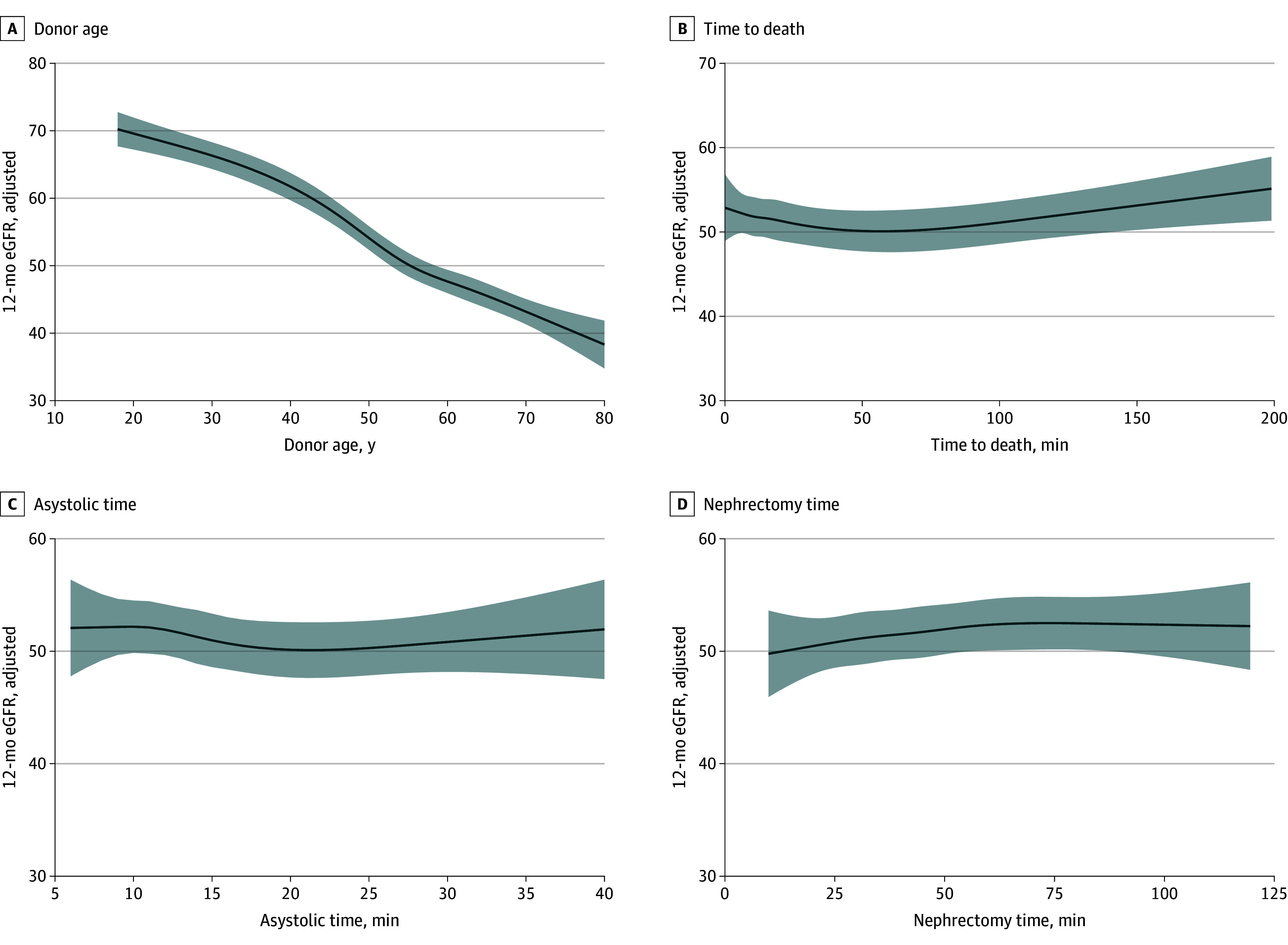
Restricted Cubic Spline Models for Variables Associated With 12-Month Posttransplant Estimated Glomerular Filtration Rate (eGFR) A, Donor age. B, Time to death (withdrawal of life-sustaining treatment to mechanical asystole). C, Asystolic time. D, Nephrectomy time. Restricted cubic splines had 5 knots and models were adjusted for all factors in Table 2. Results were pooled from 20 imputed datasets.

Restricted cubic splines allow for flexible modeling of continuous variables without assuming linearity and allows for the precise association to be assessed. As shown in Figure 2 and eFigure 2 in Supplement 1, this was performed for donor age (as the most important factor associated with outcome), TTD, and all ischemic times. This modeling confirmed that increasing TTD has no negative association with 12-month eGFR.

Functional TTD (starting at systolic blood pressure decreasing below 50 mm Hg) was analyzed in the same way as TTD and showed no association with 12-month eGFR in a standard multiple linear regression model (log_2_ fTTD coefficient, −0.04; 95% CI, −0.55 to 0.48; *P* = .89) (eTable 6 in Supplement 1) or when analyzed using the restricted cubic spline approach (eFigure 3 in Supplement 1). Most participants (estimated 7074 of 7183 [98.5%]) had an fTTD of less 30 minutes. The median fTTD was 3 minutes (IQR, 1-6 minutes) for those with TTD of less than 30 minutes, 4 minutes (IQR, 2-9 minutes) for those with TTD of 31 to 60 minutes, 4 minutes (IQR, 1-8 minutes) for those with TTD of 61 to 120 minutes, 4 minutes (IQR, 2-10 minutes) for those with TTD of 121 to 180 minutes, and 4 minutes (IQR, 2-13 minutes) for those with TTD of more than 180 minutes.

### Association With Delayed Graft Function

The association of TTD with delayed graft function was assessed using a multivariable logistic regression model, adjusting for the same set of potential confounders. As seen in eTable 7 in Supplement 1, TTD was not associated with DGF; the adjusted odds ratio (AOR) for DGF was 1.01 (95% CI, 0.97-1.06; *P* = .65) each time TTD doubled. Increasing asystolic time, CIT, and second warm ischemic time were all independently associated with increased odds of DGF (eTable 7 in Supplement 1). Nephrectomy time was not associated with increased odds of DGF. Sensitivity analyses adjusting for year of transplant, recipient hospital, machine perfusion, and highly sensitized patients did not change these conclusions, nor did addition of random intercepts for each donor.

eFigure 4 in Supplement 1 shows restricted cubic spline modeling for ischemic times associated with DGF. Again, this modeling confirmed no association of donor TTD with outcomes. These models do highlight the importance of donor age and the other ischemic times in short-term kidney transplant outcomes. There was no interaction between any of these ischemic times and NRP, suggesting that ischemic times should be minimized, even in the setting of NRP.

Functional TTD was also assessed adjusting for all factors described in eTable 7 in Supplement 1. Functional TTD was not associated with DGF in this model (AOR, 1.01; 95% CI, 0.96-1.07; *P* = .67).

### Association With Graft Survival

**Figure 3.**
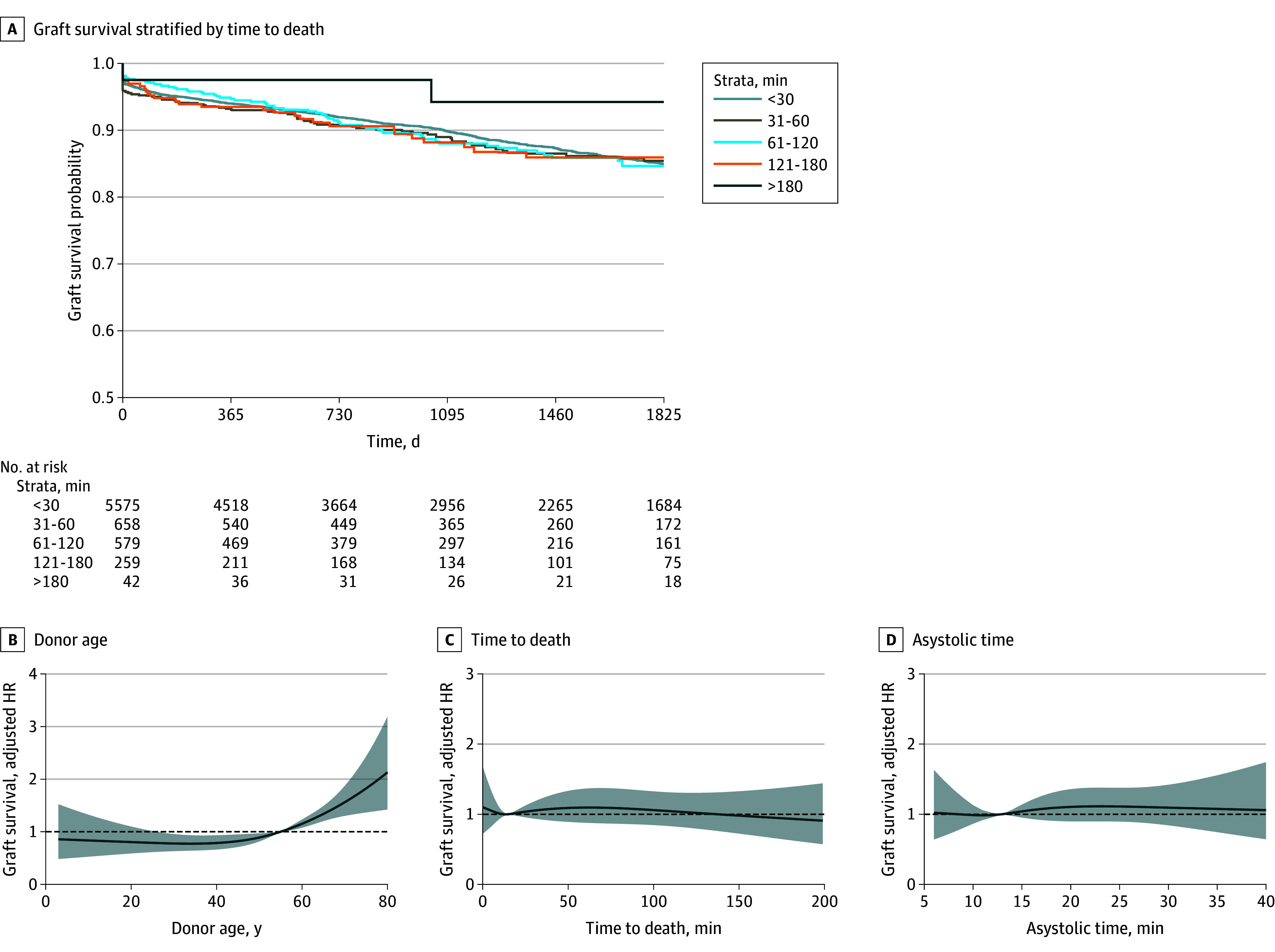
Association of Time to Death and Other Key Variables With Death-Censored Graft Survival A, Kaplan-Meier plot as a visual representation of time-to-death categories. B, Donor age. C, Time to death (withdrawal of life-sustaining treatment to mechanical asystole). D, Asystolic time. Restricted cubic splines had 4 knots and were adjusted for all factors in eTable 8 in Supplement 1. Results were pooled from 20 imputed datasets. Includes patients with complete graft survival follow-up data (n = 7113). HR indicates hazard ratio.

Figure 3A shows a Kaplan-Meier plot as a visual display of death-censored graft survival stratified by TTD. A multivariable Cox proportional hazards regression model was used to assess the association of TTD with graft survival (censored at 5 years; 799 events) (eTable 8 in Supplement 1). Time to death was not associated with this long-term outcome; the adjusted hazard ratio for graft survival was 1.00 (95% CI, 0.95-1.07; *P* = .92) each time TTD doubled. Cold ischemic time and second warm ischemic time were independently associated with graft survival, while asystolic and nephrectomy time were not (eTable 8 in Supplement 1).

All these findings were confirmed on restricted cubic spline modeling (Figure 3B-D; eFigure 5 in Supplement 1). These splines revealed the association between donor age and graft survival to be nonlinear (χ^2^_2_ = 9.69; *P* = .008). Analysis of transplant survival (graft loss or patient death) was consistent with the death-censored graft survival model regarding TTD and ischemic times (eTable 9 in Supplement 1).

### Excess Number of Transplants Based on Wait Time

As UK policy specifies a long DCD wait time of a minimum of 3 hours, the number of kidneys transplanted from donors with prolonged TTD in the UK is relevant for the many transplant settings that currently specify much shorter wait times (such as in the US).^5^ Figure 1B displays the number of transplants each year stratified by donor TTD. These data can be used to estimate the benefits associated with the UK wait time policy compared with alternative policies. Compared with a theoretical wait time of 1 hour, the UK policy has been associated with an estimated 885 extra transplants compared with 6298 transplants (14.1% increase) between 2013 and 2021. Compared with a 2-hour wait time, the UK policy has been associated 303 extra transplants compared with 6880 transplants (4.4% increase) (Figure 1B; eTable 4 in Supplement 1).

## Discussion

We have found that TTD in controlled DCD donors was not associated with recipient outcomes after kidney transplant on adjusted analyses. Specifically, there is no association with 12-month eGFR (an important surrogate for long-term graft survival),^27^ delayed graft function, or graft survival. These conclusions held when using restricted cubic splines to model nonlinear associations and on several interaction and sensitivity analyses. Therefore, kidney offers from donors with TTD of 1 to 3 hours should be evaluated using identical criteria as those used for donors with TTD less than 1 hour.

The UK is a relative outlier with a minimum DCD wait time of 3 hours, which has been UK policy since 2013.^8^ In contrast, the mean maximum wait time across US ODOs has been reported as 1 hour, 17 minutes in 2017, with only 6.7% of ODOs waiting 2 hours.^5^ We show that, by adopting a minimum wait time of 3 hours, ODOs internationally can significantly and safely increase their transplant numbers.

Existing research focusing on duration of TTD and outcomes also revealed no association of TTD duration with kidney graft survival, but there have been contrasting results regarding associations with DGF.^5,6,9^ However, these studies all have sample sizes at least 10-fold lower than the current study and frequently had maximum TTD cutoffs of 2 hours.^5,6^ Along with our use of a continuous outcome variable, this large sample size strengthens our analyses, and as such, the resulting certainty of evidence is greater.

The finding that increasing TTD (within the observed range) does not result in inferior posttransplant outcomes is also consistent with research in pancreas and liver transplants.^28,29^ This finding provides further confidence that the time between WLST and death does not cause major damage to the abdominal viscera.

Previous studies have reported warm ischemic time (from treatment WLST to start of aortic cold flush in the donor) to be an important factor in kidney transplant outcomes.^30^ However, this approach merges both TTD and asystolic time, which are physiologically distinct. Our study focused on TTD, as this is the more important factor in dictating policy regarding wait time. However, we do provide data on the ischemic time frames after mechanical asystole. We demonstrate that the deleterious associations of prolonged warm ischemic time are due to asystolic time rather than TTD. This negative association of asystolic warm ischemia is in keeping with previous work.^7,28^ We also show the association of both CIT and reperfusion time with outcomes. Therefore, efforts such as donor WLST close to the operating theater, reducing CITs through improved cross-matching tests, and optimal transplant surgical techniques should be made to reduce these times to improve recipient outcomes.^13,31^

It has been suggested that the hemodynamic profile between WLST and death may be a better variable to assess outcome than its duration. The most common approach has been to assess time from the onset of “functional” warm ischemia, usually defined by a decrease in systolic blood pressure below 50 mm Hg. A previous UK analysis reported that when this duration was over 30 minutes, there was a negative association with DGF, but not with primary nonfunction of graft survival.^32^ Our data show no association of fTTD with any of these outcomes. One explanation for this difference is that Kostakis et al^32^ did not adjust for donor factors such as donor cause of death, which are likely confounders in this setting (eTable 5 in Supplement 1). Further research has described methods for assessing trends in donor hemodynamics.^33,34^ However, these studies investigated multiple trend metrics, are prone to type I error, and require external validation.

The association of TTD beyond 3 hours with transplant outcomes is less certain. There was no trend toward worsening outcome up to 3 hours in any of our restricted cubic spline models. Also, the small cohort of kidneys from donors with TTD of more than 3 hours performed well in this study. Given these 2 findings, even if an association between TTD and inferior outcomes does exist at some time point, it is likely only relevant well beyond 3 hours.

The extent to which wait times should be increased is therefore a question of resource planning and ODO logistics. We suggest that current evidence does not support a hard cutoff of 3 hours. Decisions on when to cut off the wait time could be guided by the estimated likelihood of a donor proceeding to death. However, research predicting TTD has focused on death within 2 hours.^35,36^ A previous study has shown that among potential DCD donors not donating due to a 2-hour wait time, 20.8% died between 2 and 4 hours and 10.4% died between 4 and 6 hours.^37^ Despite long wait times, 17% of consented eligible UK DCDs did not proceed to donation due to TTD greater than 3 hours.^38^ This finding further highlights that significant reductions in unnecessary organ nonuse could be achieved by extending wait times.

### Limitations

This study has some limitations. The main limitation is the registry cohort design and inherent potential for selection bias. There is also an inevitable degree of missing data, but robust techniques were used to impute these data. In the UK, the minimum wait time of 3 hours is nationally accepted, and prolonged TTD is not seen as a barrier to transplant; therefore, significant bias will not arise from clinicians selecting kidneys based on TTD values. Also, the large sample size allowed for models adjusting for a wide range of relevant factors to limit potential selection bias.

## Conclusions

In this cohort study of recipients of a DCD kidney, donor TTD was not associated with kidney transplant outcomes. This is by far the largest study to date on the topic, to our knowledge, and included a significant number of transplants from donors with TTD over 2 hours. Our results therefore challenge ODOs and transplant services internationally, most of which have maximum wait times of 1 to 2 hours. We show that meaningful increases to transplant numbers can be safely achieved by organizations that currently implement more conservative maximum wait times. We also suggest that 3 hours should not be used as a hard cutoff, and prolonging wait time beyond 3 hours should be a balance between ODO logistics and the likelihood of proceeding.

## Notes

Supplement 1. **eTable 1.** Definitions for Key Predictor Variables **eTable 2.** Extended Demographic Variables and Missing Data Information **eTable 3.** NHS Blood and Transplant Mismatch Groups **eTable 4.** Estimated Number of Transplants per Year by Time to Death Category **eTable 5.** Cohort Demographics in Various Time to Death Groups **eTable 6.** Multiple Linear Regression Model for 12-Month eGFR, Pooled From 20 Imputed Datasets **eTable 7.** Multiple Logistic Regression Model for Delayed Graft Function, Pooled From 20 Imputed Datasets **eTable 8.** Multivariable Cox Regression Model for Death-Censored Graft Survival, Pooled From 20 Imputed Datasets **eTable 9.** Multivariable Cox Regression Model for Transplant Survival (Graft Loss or Death), Pooled From 20 Imputed Datasets **eFigure 1.** Study Flow Diagram **eFigure 2.** Restricted Cubic Spline Models for Predictors of 12-Month eGFR, Adjusted for All Factors in Table 2 **eFigure 3.** Functional Time to Death (fTTD) Model for 12-Month eGFR Adjusted for All Factors in Table 2 **eFigure 4.** Restricted Cubic Spline Models for Predictors of Delayed Graft Function, Adjusted for All Factors in eTable 7 **eFigure 5.** Restricted Cubic Spline Models for Nephrectomy Time, Cold Ischaemic Time and Reperfusion Time With Death-Censored Graft Survival, Adjusted for All Factors in eTable 8

Supplement 2.
Data Sharing Statement
