# Supplementary material for: Donor Time to Death and Kidney Transplant Outcomes in the Setting of a 3-Hour Minimum Wait Policy
Source: JAMA Netw Open. 2024 Nov 14;7(11):e2443353. doi: 10.1001/jamanetworkopen.2024.43353 (PMC11565268; doi:10.1001/jamanetworkopen.2024.43353)
Supplement: Supplement 1. — eTable 1. Definitions for Key Predictor Variables eTable 2. Extended Demographic Variables and Missing Data Information eTable 3. NHS Blood and Transplant Mismatch Groups eTable 4. Estimated Number of Transplants per Year by Time to Death Category eTable 5. Cohort Demographics in Various Time to Death Groups eTable 6. Multiple Linear Regression Model for 12-Month eGFR, Pooled From 20 Imputed Datasets eTable 7. Multiple Logistic Regression Model for Delayed Graft Function, Pooled From 20 Imputed Datasets eTable 8. Multivariable Cox Regression Model for Death-Censored Graft Survival, Pooled From 20 Imputed Datasets eTable 9. Multivariable Cox Regression Model for Transplant Survival (Graft Loss or Death), Pooled From 20 Imputed Datasets eFigure 1. Study Flow Diagram eFigure 2. Restricted Cubic Spline Models for Predictors of 12-Month eGFR, Adjusted for All Factors in Table 2 eFigure 3. Functional Time to Death (fTTD) Model for 12-Month eGFR Adjusted for All Factors in Table 2 eFigure 4. Restricted Cubic Spline Models for Predictors of Delayed Graft Function, Adjusted for All Factors in eTable 7 eFigure 5. Restricted Cubic Spline Models for Nephrectomy Time, Cold Ischaemic Time and Reperfusion Time With Death-Censored Graft Survival, Adjusted for All Factors in eTable 8 [file jamanetwopen-e2443353-s001.pdf]

Supplementary Online Content

Tingle SJ, Chung NDH, Malik AK, et al. Donor time to death and kidney transplant outcomes in the setting of a 3-hour minimum wait policy. *JAMA Netw Open.* 2024;7(11):e2443353. doi:10.1001/jamanetworkopen.2024.43353

eTable 1. Definitions for Key Predictor Variables
eTable 2. Extended Demographic Variables and Missing Data Information
eTable 3. NHS Blood and Transplant Mismatch Groups
eTable 4. Estimated Number of Transplants per Year by Time to Death Category
eTable 5. Cohort Demographics in Various Time to Death Groups
eTable 6. Multiple Linear Regression Model for 12-Month eGFR, Pooled From 20 Imputed Datasets
eTable 7. Multiple Logistic Regression Model for Delayed Graft Function, Pooled From 20 Imputed Datasets
eTable 8. Multivariable Cox Regression Model for Death-Censored Graft Survival, Pooled From 20 Imputed Datasets
eTable 9. Multivariable Cox Regression Model for Transplant Survival (Graft Loss or Death), Pooled From 20 Imputed Datasets
eFigure 1. Study Flow Diagram
eFigure 2. Restricted Cubic Spline Models for Predictors of 12-Month eGFR, Adjusted for All Factors in Table 2
eFigure 3. Functional Time to Death (fTTD) Model for 12-Month eGFR Adjusted for All Factors in Table 2
eFigure 4. Restricted Cubic Spline Models for Predictors of Delayed Graft Function, Adjusted for All Factors in eTable 7
eFigure 5. Restricted Cubic Spline Models for Nephrectomy Time, Cold Ischaemic Time and Reperfusion Time With Death-Censored Graft Survival, Adjusted for All Factors in eTable 8

This supplementary material has been provided by the authors to give readers additional information about their work.

© 2024 Tingle SJ et al. *JAMA Network Open.*

eTable 1. Definitions for Key Predictor Variables

Term	Definition
**Time to death**	Life sustaining treatment withdrawal until mechanical asystole (loss or arterial pressure wave on invasive blood pressure monitoring via arterial cannula).
**Functional time to death**	Time from systolic blood pressure falling below 50mmHg until mechanical asystole (loss or arterial pressure wave on invasive blood pressure monitoring via arterial cannula).
**Asystolic time**	Mechanical asystole (loss or arterial pressure wave on invasive blood pressure monitoring via arterial cannula) until perfusion via aortic cannula (for donors undergoing normothermic regional perfusion this is the start of NRP)
**Nephrectomy time**	Time from cold aortic cold perfusion (for donors undergoing normothermic regional perfusion, this is at the end of perfusion) to placement of the kidney graft in ice on the back table.
**Reperfusion time**	Time from kidney out of ice in the recipient hospital until reperfusion. Also called second warm ischemic time or anastomosis time.
**Cold ischemic time**	Time between aortic cold flush in the donor and kidney out of ice in the recipient hospital.

© 2024 Tingle SJ et al. *JAMA Network Open.*

eTable 2. Extended Demographic Variables and Missing Data Information. BMI = body mass index, Tac = tacrolimus, Pred = prednisolone/prednisone, MMF = mycophenolate mofetil, Aza = azathioprine.*for a description of HLA mismatch groups see eTable 3.

Donor characteristic	Patients, No. (%)
**Donor sex**	
**Male**	4476 (62.3)
**Female**	2707 (37.7)
**Missing**	0 (0.0)
**Donor age (years)**	
**Mean (SD)**	52.2 (14.6)
**Median [Min, Max]**	55.0 [3.00, 82.0]
**Donor BMI in kg/m^2**	
**Mean (SD)**	27.5 (5.78)
**Median [Min, Max]**	26.7 [10.8, 49.4]
**Missing**	47 (0.7)
**Donor ethnicity**	
**Asian**	142 (2.0)
**Black**	49 (0.7)
**Other**	126 (1.8)
**White**	6787 (94.5)
**Missing**	79 (1.1)
**Donor cause of death**	
**Hypoxic brain injury**	2847 (39.6)
**Ischemic stroke**	508 (7.1)
**Intracranial haemorrhage**	2498 (34.8)
**Trauma**	314 (4.4)
**Other**	765 (10.7)
**Missing**	251 (3.5)
**Donor creatinine at retrieval in µmol/L (log$_2$-transformed)**	
**Mean (SD)**	6.07 (0.633)
**Median [Min, Max]**	6.02 [2.58, 9.63]
**Missing**	326 (4.5)
**Cardiovascular disease status**	
**No cardiovascular disease present**	5987 (83.3)
**Cardiovascular disease present**	1024 (14.3)
**Missing**	172 (2.4)
**Donor diabetes status**	
**No diabetes present**	6533 (91.0)
**Diabetes present**	579 (8.1)
**Missing**	71 (1.0)
**Donor history of drug abuse**	
**No history of drug abuse**	5783 (80.5)
**History of drug abuse**	1245 (17.3)
**Missing**	155 (2.2)
**Donor hypertension status**	
**No hypertension present**	5022 (69.9)
**Hypertension present**	2071 (28.8)
**Missing**	90 (1.3)
**Donor smoking status**	
**Non-smoker**	2901 (40.4)
**Smoker**	4244 (59.1)
**Missing**	38 (0.5)
**Receipt of normothermic regional perfusion**	
**No**	6730 (93.7)
**Yes**	453 (6.3)
**Retrieval and transplant factors**	**Patients, No. (%)**
**Cold ischemic time in minutes**	
**Mean (SD)**	9.58 (0.520)
**Median [Min, Max]**	9.59 [4.32, 11.5]
**Missing**	24 (0.3)

© 2024 Tingle SJ et al. *JAMA Network Open.*

**Retrieval and transplant factors**	**Patients, No. (%)**
**Asystolic time in minutes**	
**Mean (SD)**	14.2 (4.23)
**Median [Min, Max]**	13.0 [6.00, 40.0]
**Missing**	2026 (28.2)
**Nephrectomy time in minutes**	
**Mean (SD)**	46.7 (20.3)
**Median [Min, Max]**	43.0 [10.0, 148]
**Missing**	1022 (14.2)
**Time to death in minutes**	
**Mean (SD)**	28.6 (38.0)
**Median [Min, Max]**	15.0 [0, 407]
**Missing**	1813 (25.2)
**Functional time to death in minutes**	
**Mean (SD)**	5.46 (11.1)
**Median [Min, Max]**	3.00 [0, 188]
**Missing**	2343 (32.6)
**Reperfusion time in minutes**	
**Mean (SD)**	39.8 (13.0)
**Median [Min, Max]**	38.0 [0, 120]
**Missing**	13 (0.2)
**Recipient characteristic**	
**Recipient sex**	
**Male**	4666 (65.0)
**Female**	2515 (35.0)
**Missing**	2 (0.0)
**Recipient age (years)**	
**Mean (SD)**	54.5 (12.8)
**Median [Min, Max]**	56.0 [18.0, 83.0]
**Recipient BMI in kg/m^2**	
**Mean (SD)**	27.4 (4.79)
**Median [Min, Max]**	27.2 [13.6, 48.7]
**Missing**	1501 (20.9)
**Recipient ethnicity**	
**Asian**	1214 (16.9)
**Black**	593 (8.3)
**Other**	152 (2.1)
**White**	5143 (71.6)
**Missing**	81 (1.1)
**Primary renal disease**	
**Diabetes**	925 (12.9)
**Glomerulonephritis**	1293 (18.0)
**Hypertension**	474 (6.6)
**Polycystic kidney disease**	933 (13.0)
**Pyelonephritis / reflux nephropathy**	391 (5.4)
**Other**	1292 (18.0)
**Missing**	1875 (26.1)
**Recipient diabetes status**	
**No diabetes present**	6258 (87.1)
**Diabetes present**	925 (12.9)
**Recipient wait time in days (Log$_2$-transformed)**	
**Mean (SD)**	9.12 (1.55)
**Median [Min, Max]**	9.46 [0, 12.3]
**Missing**	37 (0.5)
**Year of transplant**	
**Mean (SD)**	2020 (2.50)
**Median [Min, Max]**	2020 [2010, 2020]
**Number of kidney transplants**	
**First transplant**	6347 (88.4)
**Single previous transplant**	739 (10.3)
**More than one previous transplant**	97 (1.4)
**Recipient characteristic**	**Patients, No. (%)**

© 2024 Tingle SJ et al. *JAMA Network Open.*

**Calculated reaction frequency at transplant**	
**Not highly sensitised patient**	6603 (91.9)
**Highly-sensitised**	580 (8.1)
**Dialysis type at transplant**	
**Haemodialysis**	4532 (63.1)
**Peritoneal dialysis**	1443 (20.1)
**None (pre-emptive)**	26 (0.4)
**Missing**	1182 (16.5)
**Immunosuppression at transplant**	
**Tac + Pred + MMF**	5093 (70.9)
**Tac + MMF (without Pred)**	838 (11.7)
**Tac + Pred + Aza**	380 (5.3)
**Other**	852 (11.9)
**Missing**	20 (0.3)
**eGFR at 1 year in ml/min/1.73 m^2 ****	
**Mean (SD)**	48.9 (22.9)
**Median [Min, Max]**	47.4 [6.52, 129]
**Missing**	932 (13.0)
**Function of kidney post-transplant**	
**Immediate**	4333 (60.3)
**Delayed graft function**	2146 (29.9)
**Primary non-function**	192 (2.7)
**Missing**	512 (7.1)
**Graft survival at 1 year**	
**No**	419 (5.8)
**Yes**	5757 (80.1)
**Missing**	1007 (14.0)
**Perfusion quality**	
**Good**	5690 (79.2)
**Fair**	762 (10.6)
**Poor**	220 (3.1)
**Patchy**	323 (4.5)
**Missing**	188 (2.6)
**Machine perfusion:**	
**None**	6442 (89.7)
**Yes - Hypothermic**	469 (6.5)
**Yes - normothermic**	230 (3.2)
**Missing**	42 (0.6)
**HLA mismatch groups ***	
**Level 1**	238 (3.3)
**Level 2**	1801 (25.1)
**Level 3**	4092 (57.0)
**Level 4**	1052 (14.6)

© 2024 Tingle SJ et al. *JAMA Network Open.*

eTable 3. NHS Blood and Transplant Mismatch Groups

Mismatch level	Description
**1**	No mismatch
**2**	Matched for DR, 0 or 1 B mismatch, with any level of A mismatch
**3**	Either:<br>• Matched for DR with 2 B mismatches and any level of A mismatch, or<br>• 1 DR mismatch, 0 or 1 B mismatch, with any level of A mismatch
**4**	Either:<br>• 1DR mismatch and 2 B mismatches and any level of A mismatch<br>• 2 DR mismatches and any level of B/A mismatch

© 2024 Tingle SJ et al. *JAMA Network Open.*

eTable 4. Estimated Number of Transplants per Year by Time to Death Category. Estimates are pooled from all 20 imputed datasets, hence the presence of non-integers.

Time to death (minutes):	<30	31-60	61-120	121-180	>180
**Year:**					
**2013**	576.75	65.75	47.55	33.95	8
**2014**	524.2	42.6	57.2	26	3
**2015**	597.75	64.95	59.8	23.5	8
**2016**	667.55	88.8	57	33.95	4.7
**2017**	649.25	103.75	74.35	22.8	2.85
**2018**	676.1	103.95	93.4	29.35	4.2
**2019**	777.85	82.35	82.4	36.4	3
**2020**	515.75	50.5	52.5	27.25	4
**2021**	649.85	60.3	58.1	27.35	4.4

© 2024 Tingle SJ et al. *JAMA Network Open.*

eTable 5. Cohort Demographics in Various Time to Death Groups. Data averaged from all 20 imputed datasets, so that all participants are included. Continuous variables are median [IQR].

Time to death (minutes)	<30	31-60	61-120	121-180	>180
**Number of participants**	5635	663	582	261	42
**Donor age**	54 [43.0,63.0]	57 [47.0,65.0]	56 [47.0,64.0]	54 [45.0,62.0]	51 [45.0,58.0]
**Donor sex**					
Male	3561.45 (63.2%)	390.05 (58.8%)	353.95 (60.8%)	142.9 (54.8%)	27.65 (65.6%)
Female	2073.6 (36.8%)	272.9 (41.2%)	228.35 (39.2%)	117.65 (45.2%)	14.5 (34.4%)
**Donor ethnicity**					
Asian	117.25 (2.1%)	10.9 (1.6%)	8.1 (1.4%)	5.65 (2.2%)	2 (4.7%)
Black	46.4 (0.8%)	2.15 (0.3%)	1 (0.2%)	0.05 (0.0%)	0 (0%)
Other	69.9 (1.2%)	20.25 (3.1%)	33.5 (5.8%)	2.75 (1.1%)	2 (4.7%)
White	5401.5 (95.9%)	629.65 (95.0%)	539.7 (92.7%)	252.1 (96.8%)	38.15 (90.5%)
**Donor cause of death**					
Hypoxic brain injury	2203.45 (39.1%)	301.45 (45.5%)	297.75 (51.1%)	137.2 (52.7%)	16.05 (38.1%)
Ischemic stroke	454.85 (8.1%)	37.15 (5.6%)	11.4 (2.0%)	17 (6.5%)	4.15 (9.8%)
Intracranial haemorrhage	2105.75 (37.4%)	193.8 (29.2%)	193.6 (33.2%)	80.45 (30.9%)	10.45 (24.8%)
Trauma	267.7 (4.8%)	24.5 (3.7%)	22.1 (3.8%)	8.25 (3.2%)	2.05 (4.9%)
Other	603.3 (10.7%)	106.05 (16.0%)	57.45 (9.9%)	17.65 (6.8%)	9.45 (22.4%)
**Quality of cold perfusion**					
1 (good)	4619.15 (82.0%)	528.7 (79.7%)	469.05 (80.6%)	192.35 (73.8%)	30.35 (72.0%)
2 (fair)	600.7 (10.7%)	78.55 (11.8%)	60.65 (10.4%)	37.1 (14.2%)	6.5 (15.4%)
3 (poor)	163.1 (2.9%)	19.9 (3.0%)	22.25 (3.8%)	17.35 (6.7%)	5.2 (12.3%)
4 (patchy)	252.1 (4.5%)	35.8 (5.4%)	30.35 (5.2%)	13.75 (5.3%)	0.1 (0.2%)
**Normothermic regional perfusion**					
No	5305.15 (94.1%)	611.75 (92.3%)	547.15 (94.0%)	231.1 (88.7%)	34.85 (82.7%)
Yes	329.9 (5.9%)	51.2 (7.7%)	35.15 (6.0%)	29.45 (11.3%)	7.3 (17.3%)
**Cold ischaemic time**	778 [604,979]	751 [584,962]	751 [581,986]	772 [592,992]	822 [714,978]
**Recipient age**	56 [46.0,64.0]	57 [48.0,66.0]	58 [48.0,66.0]	57 [50.0,66.0]	54 [48.0,63.0]
**Recipient sex**					
Male	3663.35 (65.0%)	441.4 (66.6%)	384.45 (66.0%)	153.1 (58.8%)	24.75 (58.7%)
Female	1971.7 (35.0%)	221.55 (33.4%)	197.85 (34.0%)	107.45 (41.2%)	17.4 (41.3%)
**Recipient ethnicity**					
Asian	961.15 (17.1%)	107.65 (16.2%)	108.25 (18.6%)	47.4 (18.2%)	4.5 (10.7%)
Black	467.75 (8.3%)	51.55 (7.8%)	54.55 (9.4%)	23.25 (8.9%)	3.65 (8.7%)
Other	118 (2.1%)	12.15 (1.8%)	17.25 (3.0%)	6.25 (2.4%)	0 (0%)
White	4088.15 (72.5%)	491.6 (74.2%)	402.25 (69.1%)	183.65 (70.5%)	34 (80.7%)
**Recipient primary renal disease**					
Diabetes	711.3 (12.6%)	95.95 (14.5%)	82.65 (14.2%)	30.85 (11.8%)	4.25 (10.1%)
Glomerulonephritis	1455.95 (25.8%)	171.55 (25.9%)	138.95 (23.9%)	67.05 (25.7%)	10.15 (24.1%)
Hypertension	526.85 (9.3%)	61.85 (9.3%)	61.35 (10.5%)	23.45 (9.0%)	3.5 (8.3%)
Polycystic kidney disease	1036.1 (18.4%)	125.35 (18.9%)	108.3 (18.6%)	45 (17.3%)	14.8 (35.1%)
Pyelonephritis / reflux nephropathy	447.1 (7.9%)	52.25 (7.9%)	41.75 (7.2%)	19.15 (7.3%)	1.3 (3.1%)
Other	1457.75 (25.9%)	156 (23.5%)	149.3 (25.6%)	75.05 (28.8%)	8.15 (19.3%)
**Waitlist time**	707 [348,1170]	667 [334,1140]	722 [309,1130]	737 [321,1310]	443 [133,888]

© 2024 Tingle SJ et al. *JAMA Network Open.*

eTable 6. Multiple Linear Regression Model for 12-Month eGFR, Pooled From 20 Imputed Datasets. Functional time to death was defined as time between systolic blood pressure dropping below 50mmHg and death. Right-skewed variables are log2-transformed, so the results relate to the change in 12-month eGFR every time the variable doubles. A nominal value of eGFR 10 was used for those with graft failure before 1 year. * for HLA mismatch level details see eTable 3. TTD = Time to death (withdrawal of life sustaining treatment to mechanical asystole); NRP = Normothermic machine perfusion; BMI = Body mass index; HLA = human leukocyte antigen.

Variable	Adjusted coefficient (95% CI)	P value
**Log_2-Functional TTD**	-0.037 (-0.551 to 0.478)	0.89
**Log_2-Asystolic time**	-1.121 (-2.398 to 0.156)	0.09
**Log_2-Nephrectomy time**	0.997 (0.077 to 1.917)	0.03
**Log_2-Cold ischemic time**	-2.481 (-3.474 to -1.487)	<0.001
**Reperfusion time (per 10 minutes)**	-0.507 (-0.918 to -0.095)	0.02
**Received NRP**	5.538 (3.428 to 7.649)	<0.001
**Recipient sex: female**	-2.068 (-3.148 to -0.989)	<0.001
**Recipient age, years**	-0.085 (-0.134 to -0.035)	0.001
**Recipient BMI**	-0.321 (-0.434 to -0.208)	<0.001
**Recipient Ethnicity:**		
Asian	5.169 (3.780 to 6.559)	<0.001
Black	-6.114 (-7.983 to -4.246)	<0.001
Other	1.442 (-2.042 to 4.926)	0.42
White	Ref	
**Primary renal disease**		
Diabetes	Ref	
Glomerulonephritis	-0.888 (-2.703 to 0.927)	0.34
Hypertension	-0.220 (-2.438 to 1.997)	0.85
Polycystic kidney disease	0.676 (-1.287 to 2.638)	0.50
Pyelonephritis / Reflux nephropathy	-1.820 (-4.364 to 0.724)	0.16
Other	-1.525 (-3.318 to 0.268)	0.10
**Log_2-Recipient wait time, days**	-0.549 (-0.892 to -0.206)	0.002
**Previous kidney transplants**		
0	Ref	
1	-0.988 (-2.714 to 0.738)	0.26
>1	-7.317 (-11.47 to -3.164)	0.001
**Donor sex: Female**	-1.106 (-2.183 to -0.029)	0.04
**Donor age, years**	-0.562 (-0.607 to -0.516)	<0.001
**Donor cause of death**		
Hypoxic brain injury	Ref	
Ischemic stroke	-2.288 (-4.254 to -0.322)	0.02
Intracranial haemorrhage	-2.612 (-3.785 to -1.439)	<0.001
Trauma	0.970 (-1.531 to 3.471)	0.45
Other	-2.751 (-4.533 to -0.969)	0.003
**Log_2-Donor creatinine at retrieval, µmol/L**	-1.928 (-2.753 to -1.102)	<0.001
**Donor diabetes status: present**	-4.886 (-6.781 to -2.99)	<0.001
**Donor past history of drug abuse: present**	2.572 (1.095 to 4.049)	0.001
**Donor hypertension: present**	-3.547 (-4.731 to -2.362)	<0.001
**Visual quality of perfusion**		
Good	Ref	
Fair	-2.795 (-4.366 to -1.224)	0.001
Poor	-4.754 (-7.734 to -1.773)	0.002
Patchy	-3.379 (-5.765 to -0.993)	0.006
**HLA mismatch level***		
1	Ref	
2	-0.437 (-3.317 to 2.443)	0.77
3	0.675 (-2.106 to 3.457)	0.63
4	0.326 (-2.679 to 3.332)	0.83

© 2024 Tingle SJ et al. *JAMA Network Open.*

eTable 7. Multiple Logistic Regression Model for Delayed Graft Function, Pooled From 20 Imputed Datasets. Right-skewed variables are log2-transformed, so the results relate to the odds ratio for DGF every time the variable doubles. * for HLA mismatch level details see eTable 3. TTD = Time to death; NRP = Normothermic machine perfusion; BMI = Body mass index; HLA = human leukocyte antigen

Variable	Adjusted OR (95% CI)	P value
**Log_2-TTD**	1.01 (0.97 to 1.06)	0.65
**Log_2-Asystolic time**	1.20 (1.05 to 1.38)	0.01
**Log_2-Nephrectomy time**	1.02 (0.93 to 1.13)	0.62
**Log_2-Cold ischemic time**	1.36 (1.23 to 1.52)	<0.001
**Reperfusion time (per 10 minutes)**	1.07 (1.03 to 1.12)	0.001
**Received NRP**	0.54 (0.42 to 0.69)	<0.001
**Recipient sex: female**	0.71 (0.63 to 0.79)	<0.001
**Recipient age, years**	0.99 (0.99 to 1.00)	0.005
**Recipient BMI**	1.03 (1.01 to 1.04)	<0.001
**Recipient Ethnicity:**		
Asian	1.05 (0.91 to 1.22)	0.50
Black	1.75 (1.45 to 2.11)	<0.001
Other	0.94 (0.65 to 1.35)	0.74
White	Ref	
**Primary renal disease**		
Diabetes	Ref	
Glomerulonephritis	0.81 (0.67 to 0.98)	0.03
Hypertension	0.77 (0.61 to 0.98)	0.03
Polycystic kidney disease	0.71 (0.57 to 0.87)	0.001
Pyelonephritis / Reflux nephropathy	0.72 (0.55 to 0.94)	0.02
Other	0.78 (0.65 to 0.94)	0.009
**Log_2-Recipient wait time, days**	1.23 (1.19 to 1.28)	<0.001
**Previous kidney transplants**		
0	Ref	
1	1.52 (1.27 to 1.81)	<0.001
>1	3.77 (2.29 to 6.20)	<0.001
**Donor sex: Female**	0.84 (0.75 to 0.94)	0.003
**Donor age, years**	1.01 (1.00 to 1.01)	0.001
**Donor cause of death**		
Hypoxic brain injury	Ref	
Ischemic stroke	1.36 (1.10 to 1.69)	0.005
Intracranial haemorrhage	1.17 (1.03 to 1.33)	0.02
Trauma	0.98 (0.74 to 1.30)	0.89
Other	1.32 (1.11 to 1.58)	0.002
**Log_2-Donor creatinine at retrieval, µmol/L**	1.24 (1.13 to 1.35)	<0.001
**Donor diabetes status: present**	1.24 (1.03 to 1.49)	0.02
**Donor past history of drug abuse: present**	0.84 (0.72 to 0.99)	0.03
**Donor hypertension: present**	1.12 (0.99 to 1.27)	0.06
**Visual quality of perfusion**		
Good	Ref	
Fair	1.32 (1.12 to 1.56)	0.001
Poor	1.39 (1.03 to 1.88)	0.03
Patchy	1.36 (1.06 to 1.74)	0.02
**HLA mismatch level***		
1	Ref	
2	1.03 (0.74 to 1.41)	0.88
3	1.08 (0.79 to 1.47)	0.65
4	1.23 (0.88 to 1.72)	0.22

© 2024 Tingle SJ et al. *JAMA Network Open.*

eTable 8. Multivariable Cox Regression Model for Death-Censored Graft Survival, Pooled From 20 Imputed Datasets. Graft survival was censored at 5 years of follow up. Analysis based on the 799 events (graft loss) occurring in the 7113 patients with complete graft survival data. Right-skewed variables are log2-transformed, so the results relate to the hazard ratio for graft loss every time the variable doubles. * for HLA mismatch level details see eTable 3. TTD = Time to death; NRP = Normothermic machine perfusion; BMI = Body mass index; HLA = human leukocyte antigen.

Variable	Adjusted HR (95% CI)	P Value
**Log_2-TTD**	1.00 (0.95 to 1.07)	0.92
**Log_2-Asystolic time**	1.08 (0.92 to 1.27)	0.35
**Log_2-Nephrectomy time**	1.01 (0.89 to 1.15)	0.86
**Log_2-Cold ischemic time**	1.21 (1.05 to 1.40)	0.008
**Reperfusion time (per 10 minutes)**	1.09 (1.04 to 1.15)	0.001
**Received NRP**	0.77 (0.54 to 1.10)	0.15
**Recipient sex: female**	1.01 (0.87 to 1.17)	0.95
**Recipient age, years**	0.99 (0.98 to 0.99)	<0.001
**Recipient BMI**	1.00 (0.99 to 1.02)	0.81
**Recipient Ethnicity:**		
Asian	0.86 (0.71 to 1.05)	0.14
Black	1.21 (0.95 to 1.53)	0.12
Other	1.01 (0.61 to 1.67)	0.97
White	Ref	
**Primary renal disease**		
Diabetes	Ref	
Glomerulonephritis	0.80 (0.63 to 1.02)	0.08
Hypertension	0.73 (0.53 to 0.99)	0.05
Polycystic kidney disease	0.56 (0.41 to 0.75)	<0.001
Pyelonephritis / Reflux nephropathy	0.74 (0.53 to 1.04)	0.08
Other	0.78 (0.61 to 1.00)	0.05
**Log_2-Recipient wait time, days**	1.10 (1.05 to 1.16)	<0.001
**Previous kidney transplants**		
0	Ref	
1	1.15 (0.92 to 1.44)	0.23
>1	1.86 (1.16 to 2.98)	0.01
**Donor sex: Female**	0.98 (0.84 to 1.14)	0.75
**Donor age, years**	1.02 (1.01 to 1.02)	<0.001
**Donor cause of death**		
Hypoxic brain injury	Ref	
Ischemic stroke	1.16 (0.87 to 1.54)	0.32
Intracranial haemorrhage	1.27 (1.07 to 1.50)	0.005
Trauma	0.90 (0.60 to 1.35)	0.60
Other	1.31 (1.05 to 1.65)	0.02
**Log_2-Donor creatinine at retrieval, µmol/L**	1.17 (1.03 to 1.32)	0.01
**Donor diabetes status: present**	1.39 (1.11 to 1.74)	0.005
**Donor past history of drug abuse: present**	0.94 (0.76 to 1.17)	0.60
**Donor hypertension: present**	1.27 (1.09 to 1.48)	0.003
**Visual quality of perfusion**		
Good	Ref	
Fair	1.19 (0.96 to 1.47)	0.11
Poor	1.24 (0.86 to 1.78)	0.25
Patchy	1.16 (0.84 to 1.59)	0.36
**HLA mismatch level***		
1	Ref	
2	1.20 (0.77 to 1.88)	0.43
3	1.32 (0.85 to 2.05)	0.21
4	1.39 (0.87 to 2.23)	0.16

© 2024 Tingle SJ et al. *JAMA Network Open.*

eTable 9. Multivariable Cox Regression Model for Transplant Survival (Graft Loss or Death), Pooled From 20 Imputed Datasets. Censored at 5 years of follow up. Analysis based on the 1399 events occurring in the 7118 patients with complete graft or patient survival data. Right-skewed variables are log2-transformed, so the results relate to the hazard ratio for graft loss every time the variable doubles. * for HLA mismatch level details see eTable 3. TTD = Time to death; NRP = Normothermic machine perfusion; BMI = Body mass index; HLA = human leukocyte antigen.

Variable	Adjusted HR (95% CI)	P value
**Log_2-TTD**	0.99 (0.95 to 1.04)	0.75
**Log_2-Asystolic time**	1.06 (0.93 to 1.20)	0.42
**Log_2-Nephrectomy time**	1.03 (0.93 to 1.14)	0.53
**Log_2-Cold ischemic time**	1.18 (1.06 to 1.31)	0.003
**Reperfusion time (per 10 minutes)**	1.08 (1.04 to 1.13)	<0.001
**Received NRP**	0.82 (0.63 to 1.09)	0.17
**Recipient sex: female**	0.95 (0.85 to 1.07)	0.40
**Recipient age, years**	1.01 (1.01 to 1.02)	<0.001
**Recipient BMI**	1.00 (0.98 to 1.01)	0.47
**Recipient Ethnicity:**		
Asian	0.96 (0.82 to 1.11)	0.55
Black	1.11 (0.91 to 1.35)	0.29
Other	0.89 (0.59 to 1.36)	0.60
White	Ref	
**Primary renal disease**		
Diabetes	Ref	
Glomerulonephritis	0.74 (0.62 to 0.89)	0.001
Hypertension	0.67 (0.52 to 0.87)	0.002
Polycystic kidney disease	0.55 (0.44 to 0.69)	<0.001
Pyelonephritis / Reflux nephropathy	0.66 (0.50 to 0.87)	0.003
Other	0.73 (0.61 to 0.88)	0.001
**Log_2-Recipient wait time, days**	1.10 (1.06 to 1.14)	<0.001
**Previous kidney transplants**		
0	Ref	
1	0.74 (0.59 to 0.92)	0.006
>1	1.33 (0.84 to 2.11)	0.23
**Donor sex: Female**	1.02 (0.90 to 1.15)	0.77
**Donor age, years**	1.01 (1.01 to 1.02)	<0.001
**Donor cause of death**		
Hypoxic brain injury	Ref	
Ischemic stroke	1.03 (0.83 to 1.29)	0.78
Intracranial haemorrhage	1.14 (1.00 to 1.29)	0.05
Trauma	0.90 (0.66 to 1.22)	0.48
Other	1.15 (0.96 to 1.37)	0.13
**Log_2-Donor creatinine at retrieval, µmol/L**	1.07 (0.97 to 1.18)	0.15
**Donor diabetes status: present**	1.21 (1.01 to 1.45)	0.04
**Donor past history of drug abuse: present**	0.99 (0.83 to 1.18)	0.95
**Donor hypertension: present**	1.23 (1.09 to 1.38)	0.001
**Visual quality of perfusion**		
Good	Ref	
Fair	1.13 (0.96 to 1.34)	0.14
Poor	1.09 (0.81 to 1.47)	0.56
Patchy	1.04 (0.81 to 1.35)	0.74
**HLA mismatch level***		
1	Ref	
2	1.12 (0.79 to 1.6)	0.52
3	1.21 (0.86 to 1.70)	0.29
4	1.27 (0.88 to 1.82)	0.21

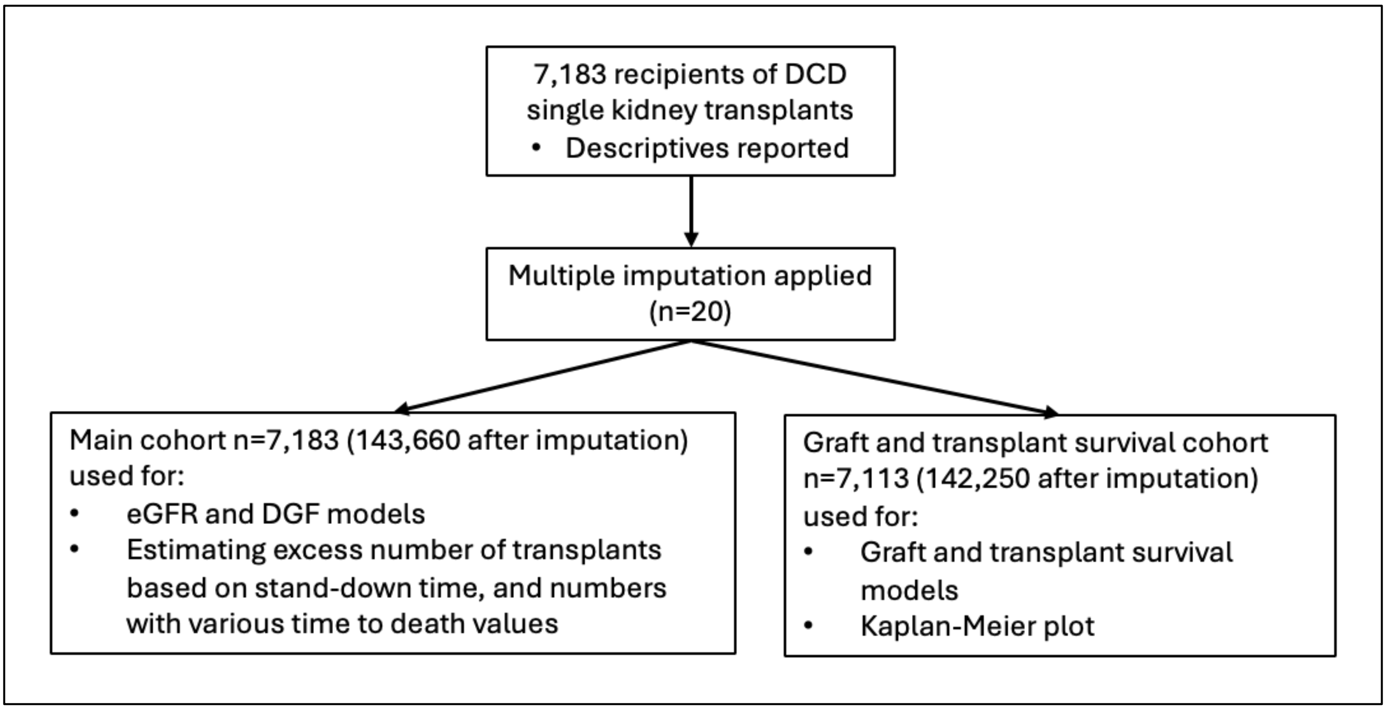

eFigure 1. Study Flow Diagram

© 2024 Tingle SJ et al. *JAMA Network Open.*

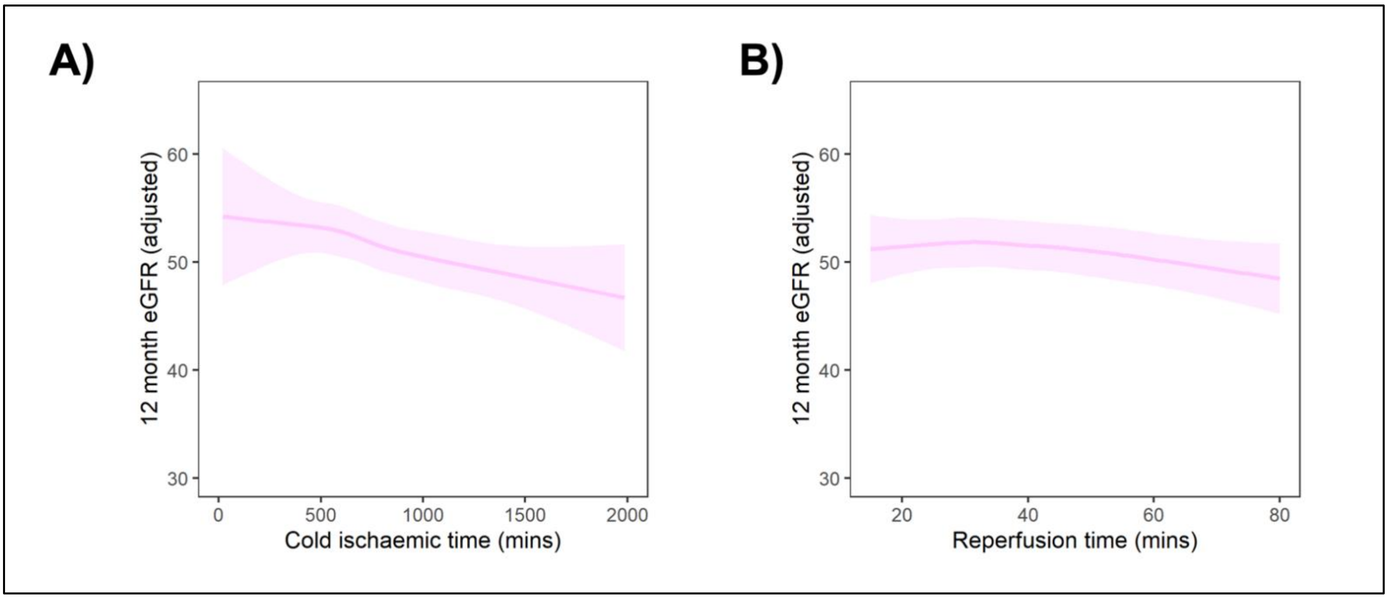

eFigure 2. Restricted Cubic Spline Models for Predictors of 12-Month eGFR, Adjusted for all Factors in Table 2. A) cold ischemic time and B) reperfusion time plotted against 12-month post-transplant estimated glomerular filtration rate (eGFR). Pooled results from 20 imputed datasets, restricted cubic splines with 5 knots.

© 2024 Tingle SJ et al. *JAMA Network Open.*

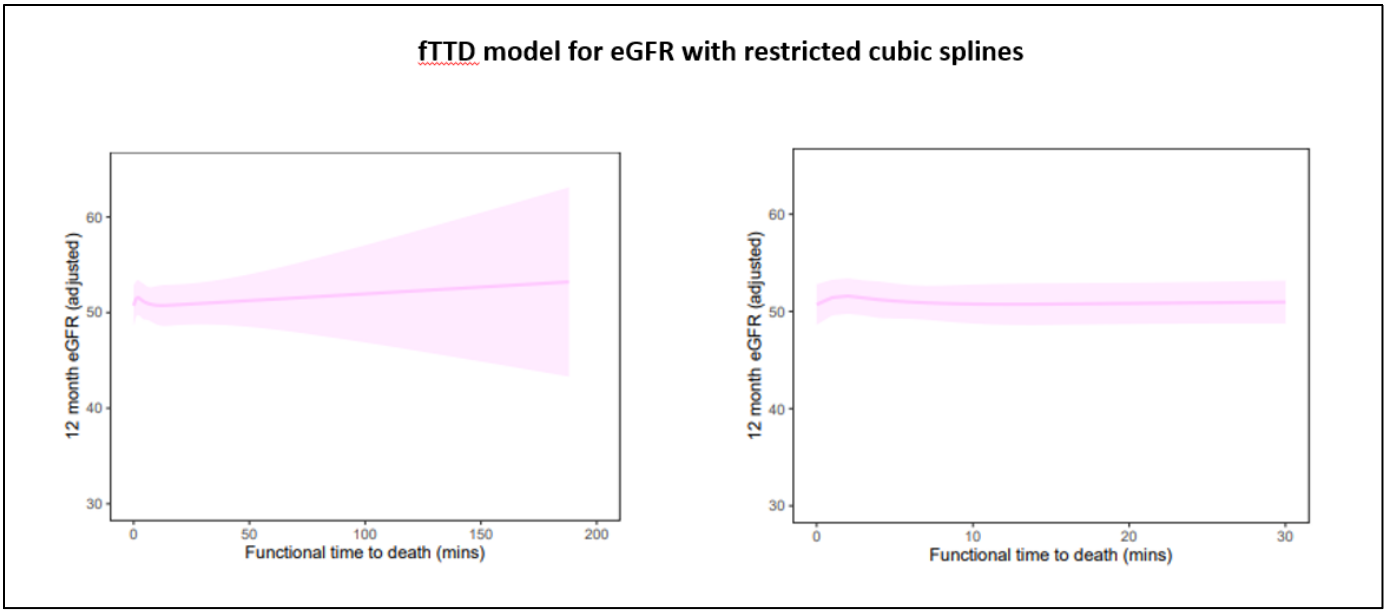

eFigure 3. Functional Time to Death (fTTD) Model for 12-Month eGFR Adjusted for all Factors in Table 2. Second panel shows functional time to death cut to the first 30 minutes as the vast majority of fTTD is less than 30 minutes. Pooled results from 20 imputed datasets, restricted cubic splines with 5 knots.

© 2024 Tingle SJ et al. *JAMA Network Open.*

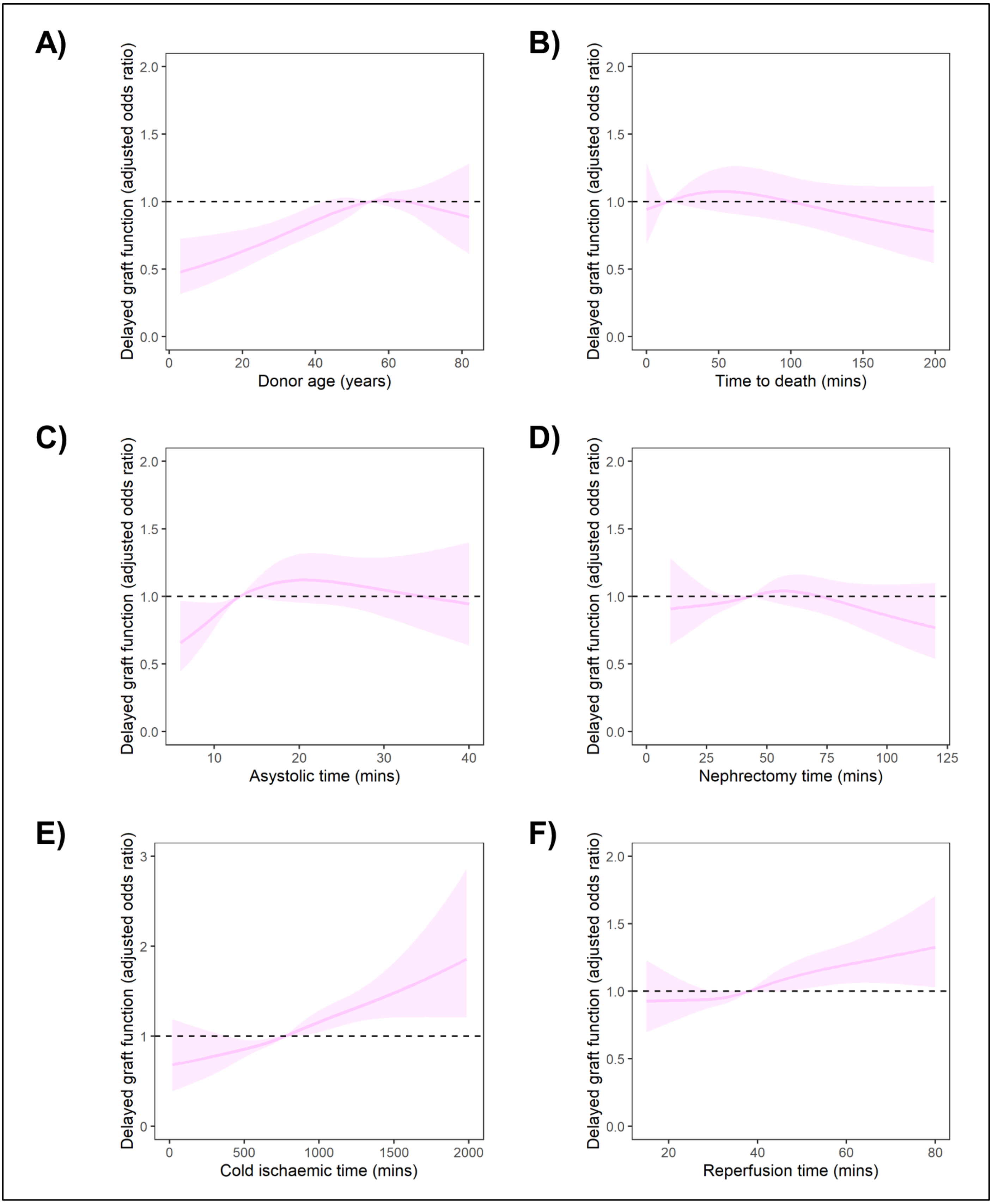

eFigure 4. Restricted Cubic Spline Models for Predictors of Delayed Graft Function, Adjusted for all Factors in eTable 7. A) donor age, B) time to death, C) asystolic time, D) nephrectomy time, E) cold ischemic time and F) reperfusion time plotted against adjusted odds ratio for DGF. Pooled results from 20 imputed datasets, restricted cubic splines with 5 knots.

© 2024 Tingle SJ et al. *JAMA Network Open.*

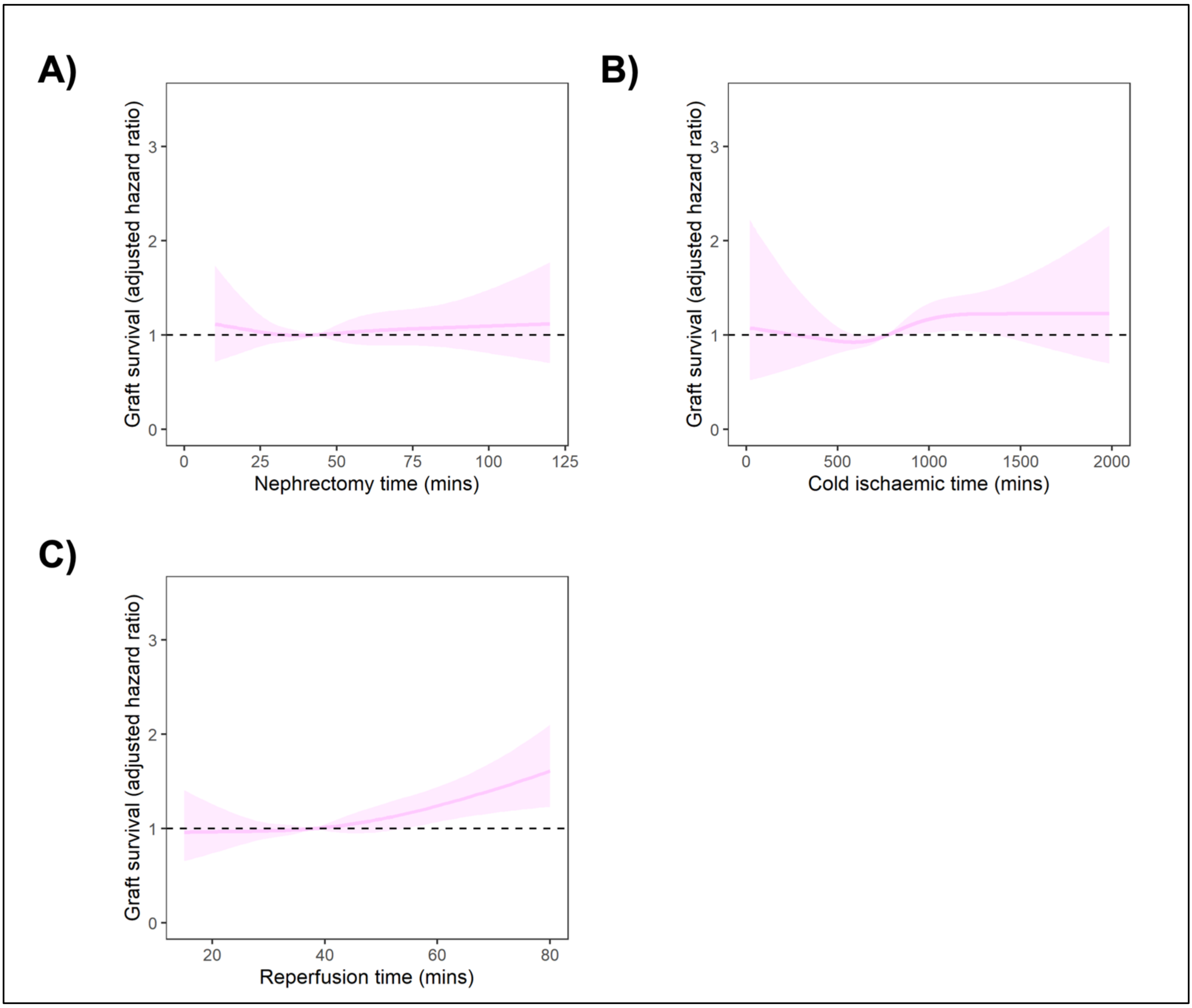

eFigure 5. Restricted Cubic Spline Models for Nephrectomy Time, Cold Ischaemic Time and Reperfusion Time With Death-Censored Graft Survival, Adjusted for all Factors in eTable 8. A) nephrectomy time, B) cold ischemic time and C) reperfusion time plotted against adjusted hazard ratio for graft loss. Pooled results from 20 imputed datasets, restricted cubic splines with 4 knots. Includes patients with complete graft survival follow up data (n=7113).

© 2024 Tingle SJ et al. *JAMA Network Open.*
