# Supplementary material for: Donor Time to Death and Kidney Transplant Outcomes in the Setting of a 3-Hour Minimum Wait Policy
Source: JAMA Netw Open. 2024 Nov 14;7(11):e2443353. doi: 10.1001/jamanetworkopen.2024.43353 (PMC11565268; doi:10.1001/jamanetworkopen.2024.43353)
Supplement: Supplement 2. — Data Sharing Statement [file jamanetwopen-e2443353-s002.pdf]

Data Sharing Statement

Tingle. Donor Time to Death and Kidney Transplant Outcomes in the Setting of a 3-Hour Minimum Wait Policy. *JAMA Netw Open*. Published November 14, 2024. doi:10.1001/jamanetworkopen.2024.43353

Data
Data available: No

Additional Information
Explanation for why data not available: The data used in this manuscript is managed by the United Kingdom Transplant Registry through NHS Blood and Transplant (NHSBT). The authors are not able to provide the raw data, however this may be requested from NHSBT through written request.
